# Cluster and Factor Analysis of Elements in Serum and Urine of Diabetic Patients with Peripheral Neuropathy and Healthy People

**DOI:** 10.1007/s12011-019-01747-x

**Published:** 2019-05-28

**Authors:** Wenjia Guo, Qi Zhou, Yanan Jia, Jiancheng Xu

**Affiliations:** 1 Department of Laboratory Medicine, First Hospital of Jilin University, Changchun, China; 2 Department of Pediatrics, First Hospital of Jilin University, Changchun, China

**Keywords:** Elements, Diabetic peripheral neuropathy, Multivariate analysis, Factor analysis, Cluster analysis

## Abstract

Diabetic peripheral neuropathy (DPN) is a common complication of diabetes mellitus, presented as a major teratogenic cause worldwide. This study discussed alternation and correlation of magnesium (Mg), calcium (Ca), copper (Cu), zinc (Zn), iron (Fe), chromium (Cr), and selenium (Se) among DPN patients and healthy people using multivariate statistical analysis. Fifty patients with DPN were recruited from endocrinology department, First Hospital of Jilin University between January 2010 and October 2011 and also 50 healthy subjects were enrolled at the same time. Inductively coupled plasma mass spectrometry (ICP-MS) was used to assay elements in serum and urine. Cluster analysis was used to clarify alternation of elements’ homogeneity. Factor analysis was performed to evaluate the most informative kinds of elements. Mg, Ca, Zn, and Cr in DPN patients were significantly lower in serum whereas significantly higher in urine. Elements were clustered into 4 or 5 clusters based on internal association using between-groups linkage algorithm. Serum Cr, Se, and Fe were grouped, and Mg related to Ca more closely in both serum and urine in DPN. Factor analysis revealed discrepancies of elements’ contribution. Cr, Se, and Fe appeared to be the most crucial factors contributing to DPN. Mg, Ca, Zn, and Cu were more influential, whereas Cr became less potent to disease. Contributed value of elements could be determined and specified using loadings in scree plot. Future studies and delicate statistical models should be applied.

## Introduction

Diabetic peripheral neuropathy (DPN) is a common complication of diabetes mellitus [1], and about 50% patients are suffering from it [2, 3]. Some scholars predict that 10% or so newly diagnosed diabetes patients are with DPN [4], disorders presented as the major teratogenic cause worldwide [5]. Besides symptoms such as pain and sensory deprivation, patients with DPN may suffer from ulcers at the extremities [6, 7]. It is a primary cause of amputation among the diabetic population [6] and damages their quality of life consequently.

Recent studies have confirmed that disturbed element metabolism under glucose disorders interact with diabetes progression, complication occurrence, and vice versa [7]. Different status as prediabetes, different types and diverse complications exert unlike changes of elements [8, 9]. Elements relate to each other closely [10], and it is very likely that information be missing or even misbelieved when dissociate just one of them. Therefore, classification methods could aggregate certain elements which contain similar information and, meanwhile, identify some of the most effective ones that may efficiently refine content and be time and cost saving.

It is stated that many elements alter homeostasis through various mechanisms. Ca affects insulin secretion of β-cells, and elevation of intracellular calcium promotes type 2 diabetes mellitus complications [11]. Mg is a key cofactor for many enzymes in carbohydrate metabolism and improves insulin resistance [12]. High concentration of Fe damages pancreatic β-cells [13]. Se prevents diabetes complications by selenium-dependent glutathione peroxidase and other selenoproteins involved in antioxidant system [14]. Except being essential for carbohydrate metabolism, Cr is cofactor for insulin action as well and helps to maintain glucose homeostasis [15]. Zn participates in signaling of insulin receptor initiation and regulation of its synthesis [16]. Cu has insulin-like activity and promotes lipogenesis [17].

The purpose of this study is to compare Mg, Ca, Cu, Zn, Se, Cr, and Fe levels between DPN patients and healthy ones, which are the most studied and “powerful” ones [18]. Cluster analysis is used to classify elements, and elements with greater potency are observed in factor analysis. Previous research of our group [8, 9] found and proved these substances altered in both blood and urine, supposing that their metabolic rate and mode were different in diabetes. Thus, we conduct this experiment of two fluids. We investigated imbalance characteristics of elements in serum and urine of patients with DPN using cluster analysis and factor analysis.

## Materials and Methods

### Subjects

This experiment was based on previous studies [8]. In brief, 50 patients with DPN (29 males and 21 females, ages range of 27–79 with median age of 56 years old, duration of diabetes 7 ± 2.5 years, BMI 21.56 ± 3.65 kg/m^2^, serum glucose 9.40 ± 3.31 mmol/L, hemoglobin A_1c_ 8.06 ± 1.68%) were enrolled to endocrinology department of the First Hospital of Jilin University from January 2010 to October 2011, and 50 healthy subjects (31 males and 19 females, ages of 20–59 years old with median age of 50 years old, BMI 25.03 ± 3.07 kg/m^2^, serum glucose 4.62 ± 1.23 mmol/L, hemoglobin A_1c_ 4.15 ± 1.24%) were examined and tracked due to their electrical record in the same period. All participants had signed the informed statement. DPN patients were affirmed that not taking element supplementation and vitamins. Their liquid intake was approximately 2000 ± 500 ml, including 250~500 ml intravenous injection drugs, 250 ml sugar-free milk, 500~1000 g vegetables, and drinking water. Their baseline characteristics were detailed described and adjusted by covariance analysis to control confounding factors. The selection criteria were based on reliable diagnosis from electronic medical record. The diagnostic standard referred to *Diagnostic criteria for diabetic peripheral neuropathy* by Chinese Doctor Association (2009) [19]: (1) detailed history and of diabetes; (2) signs and symptoms: distal symmetric polyneuropathy, focal mononeuropathy, asymmetric multiple focal neuropathy, multiple radiculopathy, and autonomic neuropathy. This study was approved by the institutional ethics committee of the First Hospital of Jilin University (2009-004) and conformed to the provisions of the Declaration of Helsinki.

### Measurement

The procedure was standardized, the same as previous experiments [8, 20, 21]. Blood and morning urine samples obtained from subjects were taken after fasting for at least 8 h into special metal-free tubes (pre-soaked in 20% *v*/*v* nitric acid, 72 h) for analysis of elements. Separated serum was preserved; 10 ml urine was conserved from each urine sample. All samples were frozen at − 80 °C before detection. Inductively coupled plasma mass spectrometry (ICP-MS) was performed by an experienced technician for determination of Mg, Ca, Cu, Zn, Fe, Cr, and Se in serum and urine. Quality control of samples was conducted with standard reference materials from China standard Material Center. Limits of detection were 1.0 μg/L for Cr, Fe, Cu, Zn, and Se while 10.0 μg/L for Mg and Ca. Recovery of standard trace elements (accuracy) ranged from 93.0 to 98.9%.

### Statistical Analysis

Shapiro-Wilk test was performed on continuous data to determine normality. Mean ± standard deviation ($\overline{x}$±s) or median (percentile) [*M* (P_25_~P_75_)] was selected for description. Comparison between groups was performed applying independent sample *t* test or Mann-Whitey *U* test. Pearson correlation was used for the linear dependence between two continuous variables, and the Spearman correlation was chosen for nonparametric measure of association between two variables.

Hierarchical cluster analysis was adopted after using the *Z* score method for data process. The correlation coefficient is compared by between-groups linkage algorithm, displaying as dendrogram. The interpretation of the diagram can be determined by cluster number based on discrepancy among categories and logical explanation. For clear observation, a line could be drawn at the extent for classification of selected cluster and corresponding variables under this category on axis. The smaller the value on the vertical axis as parting, the higher homogeneity variables own [10]. Detailed theory refers to production [22–24].

KMO and Bartlett’s spherical test were performed to evaluate applicability of factor analysis on condition of KMO value > 0.5 and Bartlett test *p* < 0.05. The first three factors are extracted after rotating with maximum variance method. Loadings were calculated and scree plots were drawn. Scree plot is a useful figure in geology, the horizontal axis stands for factors, and the vertical axis shows eigenvalue. The eigenvalue, representing information equal to how many variables have explained, is regarded as a measurement of factors’ weight. Each point means eigenvalue of corresponding factor. Proportion of containing information of the first three factors is indicated.

Statistical analysis and graphing were completed using SPSS 24.0.

## Results

### Comparison of Elements Concentration in Serum and Urine Between Control and DPN Group

As shown in Table 1, except urinary copper (UCu), serum Fe, urinary iron (UFe), serum Se, and urinary selenium (USe), other indicators had shown significant differences between two groups. The levels of Mg, Ca, Zn, and Cr in serum of DPN patients were significantly lower than those of control (*p* < 0.05), while these elements in urine as well as serum Cu concentration were significantly higher in control (*p* < 0.05).

**Table 1:** Comparison of element concentrations in serum and urine between control and DPN patients

	CON (*n* = 50)	DPN (*n* = 50)	*p*
Mg (mg/L)	35.71 (30.78~39.72)	21.62 (15.81~28.63)	< 0.001*
UMg (mg/L)	34.37 ± 22.45	61.09 ± 20.70	< 0.001*
Ca (mg/L)	164.95 (145.83~177.22)	92.57 ± 40.93	< 0.001*
UCa (mg/L)	62.04 ± 36.73	95.50 ± 49.78	< 0.001*
Cu (mg/L)	0.844 ± 0.159	1.129 ± 0.260	< 0.001*
UCu (mg/L)	0.029 ± 0.008	0.033 ± 0.010	0.066
Zn (mg/L)	0.803 ± 0.124	0.646 ± 0.114	< 0.001*
UZn (mg/L)	0.240 ± 0.102	0.553 ± 0.140	< 0.001*
Cr (mg/L)	0.185 (0.136~0.221)	0.135 (0.073~0.173)	0.001*
UCr (mg/L)	0.034 (0.024~0.041)	0.046 ± 0.025	0.005*
Fe (mg/L)	1.167 (1.402~2.185)	1.841 (1.147~2.409)	0.923
UFe (mg/L)	0.410 ± 0.239	0.491 ± 0.330	0.138
Se (mg/L)	0.067 (0.056~0.084)	0.064 (0.046~0.090)	0.363
USe (mg/L)	0.017 (0.012~0.023)	0.014 ± 0.008	0.083

*p < 0.05, there is statistically significant difference

### Correlation Analysis of Element Concentrations in Serum and Urine in Control and DPN Group

An important requirement for factor analysis application is multicollinearity among variables, i.e., relationship to each other in certain way. The closer relationship variables present, the better the dimension reduction effect is. Table 2 showed that there was correlation between elements in serum and urine. Besides, whether the correlation was statistically significant and coefficients had changed under the DPN group.

**Table 2:** Correlation coefficients of elements in serum and urine in control and DPN patients

	FBG	Mg	UMg	Ca	UCa	Cu	UCu	Zn	UZn	Cr	UCr	Fe	UFe	Se	USe
CON															
FBG	1														
Mg	0.126	1													
UMg	− 0.079	− 0.149	1												
Ca	0.237	0.192	− 0.061	1											
UCa	− 0.075	− 0.071	0.491^*^	− 0.104	1										
Cu	− 0.179	0.099	− 0.210	0.125	− 0.071	1									
UCu	− 0.197	0.077	0.325^*^	− 0.072	0.311^*^	− 0.117	1								
Zn	− 0.162	0.078	− 0.013	0.074	− 0.042	0.296^*^	− 0.138	1							
UZn	− 0.088	− 0.026	0.323^*^	− 0.152	0.208	− 0.009	0.037	− 0.059	1						
Cr	− 0.170	0.189	− 0.030	− 0.071	0.175	0.366*	0.027	0.301*	− 0.111	1					
UCr	− 0.063	− 0.069	0.609*	− 0.228	0.530*	− 0.220	0.586*	− 0.162	0.321*	0.096	1				
Fe	− 0.482*	− 0.132	0.026	− 0.220	0.081	0.490*	0.013	0.360*	− 0.094	0.419*	− 0.116	1			
UFe	− 0.113	− 0.001	0.347^*^	− 0.041	0.545^*^	0.035	0.644^*^	− 0.052	0.110	0.370^*^	0.673^*^	− 0.007	1		
Se	− 0.216	0.027	0.011	0.142	− 0.046	0.447*	− 0.083	0.352*	− 0.036	0.526*	0.006	0.405*	0.157	1	
USe	− 0.084	− 0.142	0.384*	− 0.062	0.587*	0.004	0.193	− 0.277	0.189	− 0.078	0.537*	− 0.045	0.233	− 0.023	1
DPN															
FBG	1														
Mg	0.180	1													
UMg	0.128	− 0.305^*^	1												
Ca	0.123	0.676*	− 0.151	1											
UCa	0.103	− 0.143	0.677^*^	− 0.112	1										
Cu	0.319^*^	0.001	0.176	− 0.084	0.128	1									
UCu	0.046	− 0.137	0.434^*^	− 0.045	0.268	0.239	1								
Zn	− 0.111	0.012	0.061	0.073	− 0.004	0.237	0.081	1							
UZn	0.196	− 0.144	0.459^*^	− 0.230	0.428^*^	0.351^*^	0.390^*^	− 0.051	1						
Cr	0.115	− 0.128	0.126	0.144	− 0.004	0.190	− 0.013	0.115	− 0.148	1					
UCr	0.185	− 0.025	0.302^*^	− 0.038	0.166	0.220	0.39^*^	0.131	0.226	0.127	1				
Fe	− 0.159	− 0.009	0.064	− 0.003	− 0.052	0.279	− 0.045	0.274	− 0.062	0.50*	0.127	1			
UFe	0.069	− 0.096	0.543^*^	− 0.012	0.664^*^	0.035	0.341^*^	− 0.003	0.359^*^	− 0.138	0.182	0.017	1		
Se	0.092	− 0.017	0.116	− 0.028	0.038	0.410*	0.076	0.187	− 0.028	0.512*	0.297*	0.649*	− 0.089	1	
USe	− 0.055	− 0.299^*^	0.272	− 0.352^*^	0.293^*^	0.154	0.351^*^	0.114	0.297^*^	− 0.201	0.369^*^	− 0.190	0.288^*^	0.077	1

*p < 0.05, there is statistically significant difference

### Cluster Analysis of Element Concentrations in Serum and Urine in Control and DPN Group

**Fig. 1:**
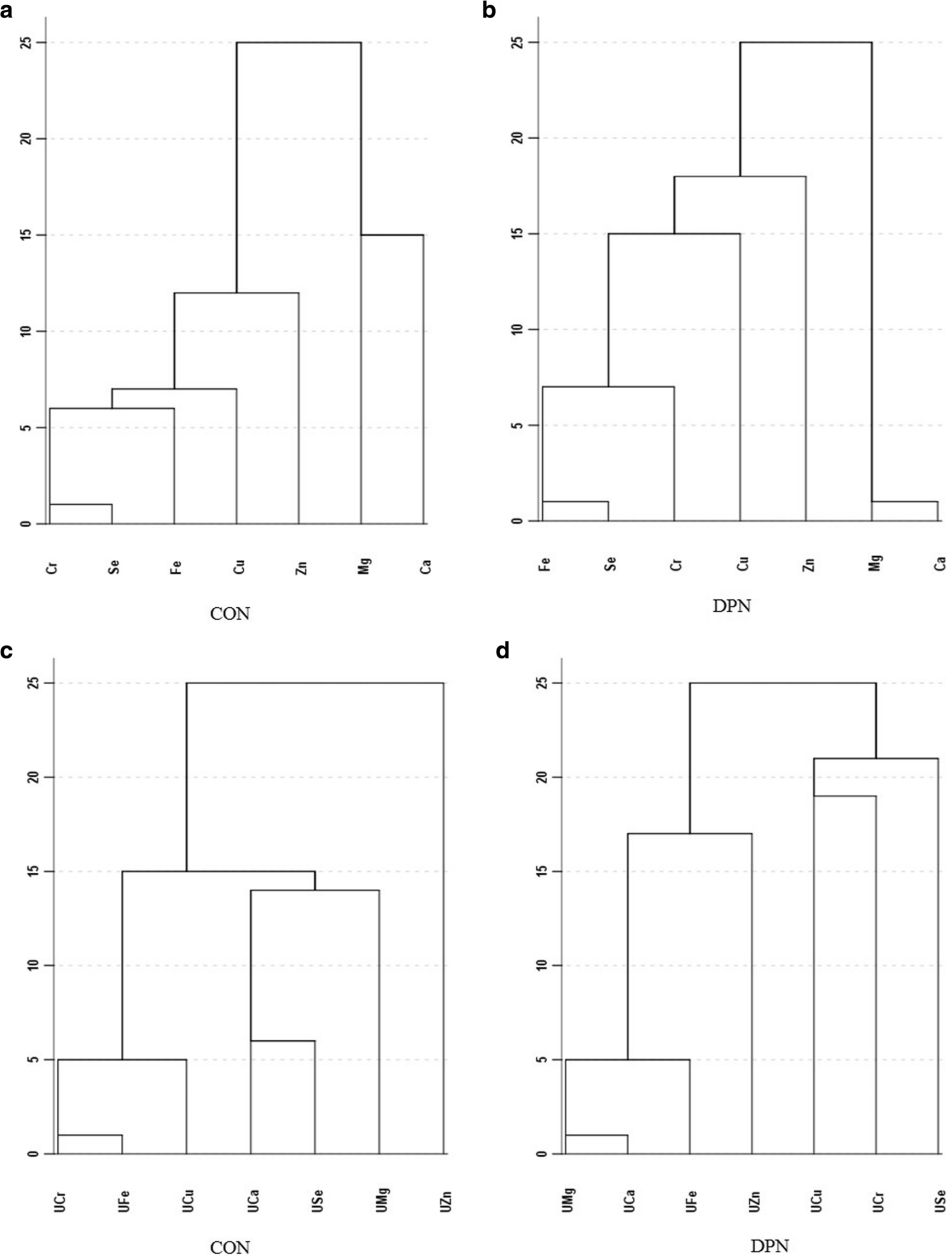
Dendrogram from cluster analysis of trace elements in serum of control (**a**) and DPN group (**b**) showing their division into four to five clusters at 10 and the same as those in the urine of control (**c**) and DPN group (**d**), respectively. U, Urinary

Figure 1 a, b, and c were grouped into 4 clusters with Fig. 1d into 5 at 10. The clusters of serum elements in control were Cr, Se, Fe, Cu, Zn, Mg, and Ca; clusters of serum elements in DPN group were Cr, Se, Fe; Cu; Zn; Mg, and Ca. Urinary elements in control were Cr, Fe, Cu; Ca, Se, Mg, and Zn; urinary elements in DPN group are Mg, Ca, Fe, Zn, Cu, Cr, and Se, respectively. It is obvious that serum Cr, Se, and Fe were clustered in both two groups. Association of Mg and Ca both in serum and urine of the DPN group were closer whereas serum Cu was weakly related to other elements, the same as what urinary Cr and Se were with the other.

### Factor Analysis of Elements in Serum and Urine in Control and DPN Group

**Fig. 2:**
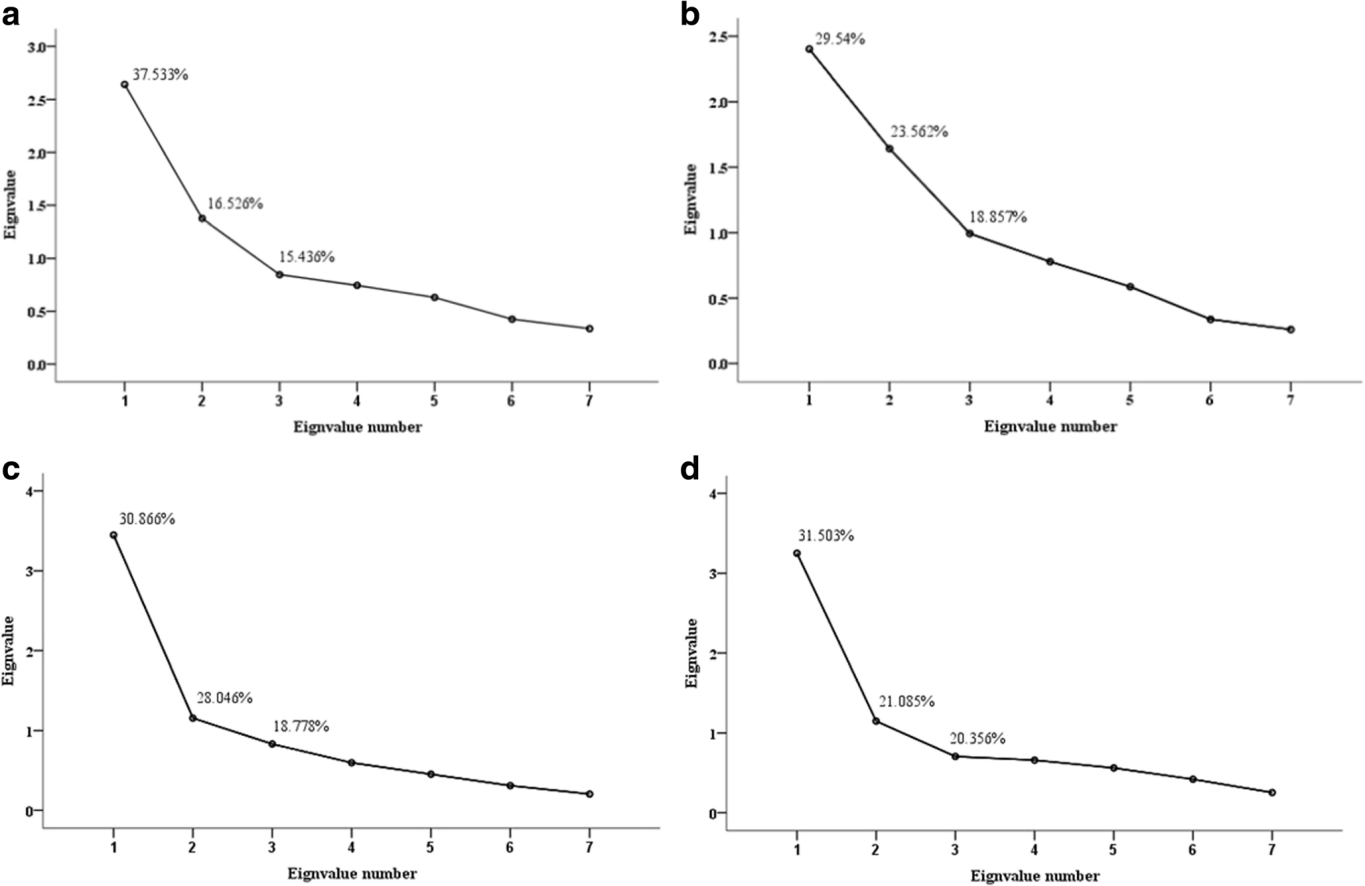
Eigenvalues of the correlation matrix. The first three factors representing percentage of information in serum of control (**a**) and DPN group (**b**), the same as those in the urine of control (**c**) and DPN group (**d**), respectively

The first 3 eigenvalues-factors containing the most information of models-were extracted and described throughly in Fig. 2. Loadings, the correlation coefficient between each element and the primary 3 factors extracted, were shown in Table 3. The value beyond 0.5 is interpreted as relatively strong relevance.

The levels of Mg, Ca, Zn, and Cr in urine and serum Cu in the DPN group were significantly higher. Various experiments resulted differently, Zhu [63] found that elements in urine of the DPN group were lower than control while this was not quite accurate on multivariable analysis stating only 24 h urine volume was different between two groups. Gómez T [64] concluded that concentration of Zn and Ca in urine of T1D rats was higher than those of control. However, when they applied 24 h urine specimens, the conclusion was just the opposite. They believed that this phenomenon was due to 24 h urine volume discrepancy (*r* > 0.7, *p* < 0.001). Therefore, to explore difference of urine elements more accurately, 24 h urine specimen should be optimal.

**Table 3:** Loadings of variables on three principal components of elements in control and DPN patients

Element	CON						DPN					
	Serum			Urinary			Serum			Urinary		
	F1	F2	F3	F1	PC2	PC3	PC1	PC2	PC3	PC1	PC2	PC3
Mg	0.022	0.138	*0.960*	0.343	0.413	*0.518*	− 0.105	*0.899*	0.071	*0.700*	0.454	0.135
Ca	0.074	*0.939*	0.141	0.338	*0.783*	0.130	0.175	*0.903*	− 0.076	*0.896*	0.170	0.088
Cu	*0.733*	0.183	0.021	0.908	0.037	0.029	0.258	− 0.104	*0.678*	0.123	*0.740*	0.377
Zn	*0.595*	0.095	0.103	− 0.017	0.078	*0.946*	0.047	0.092	*0.822*	0.350	*0.744*	0.030
Cr	*0.736*	− 0.322	0.270	*0.626*	0.524	0.345	*0.838*	0.054	− 0.077	− 0.014	0.350	*0.744*
Fe	*0.728*	− 0.321	− 0.221	*0.843*	0.300	0.050	*0.790*	0.051	0.297	*0.833*	0.134	0.158
Se	*0.812*	0.082	− 0.076	0.047	*0.899*	0.112	*0.794*	− 0.030	0.283	0.282	0.014	*0.823*
Eigenvalue	2.627	1.157	1.080	2.161	1.963	1.314	2.068	1.649	1.320	2.205	1.476	1.425
% of variance explained	37.533	16.526	15.436	30.866	28.046	18.778	29.540	23.562	18.857	31.503	21.085	20.356
% cumulative variance	37.533	54.058	69.495	30.866	58.912	77.690	29.540	53.101	71.959	31.503	52.588	72.994

Significant correlation coefficients (> 0.05) are italicized. *F* Factor

## Discussion

### Alternation in Elements of Serum and Urine of Control and DPN Group

Trace elements are emerging hotspots in the field of diabetes. With deepening of research and achievement transformation, new perspectives were provided for clarification of mechanism and treatment. There are clinical trials and experimental complementary therapies now.

In this experiment, serum Mg, Ca, Zn, and Cr in the DPN group were significantly lower than those of control, which was consistent with the discoveries of our team [8, 9] and other scholars [18, 25, 26]. Mg is considered to be an independent predictor of type 2 diabetes progression [25, 27, 28] and its complications [8, 29, 30], and clinical experiments have confirmed that Mg supplementation can improve hyperglycemia, oxidative stress, and inflammatory response in diabetic patients [31]. Zhang [7] and other investigators [18, 32, 33] demonstrated that serum Mg was lower in patients with diabetes, and Zhang [7] found that Mg was associated with parameters reflecting nerve conduction efficacy in DPN patients [7]. It has been stated that diabetes is characterized with negative Mg balance [20], for reduced gastrointestinal absorption [34], altered renal reabsorption [35], and redistribution caused by metabolic and/or pH disorders [35]. Though it’s still controversial, certain basic and clinical trial have already witnessed that Mg supplementation improved diabetes. It might protect diabetic nephropathy [36] by improving glycemic control [31] and repairing tubular injury [37] and exerting negative effect on insulin sensitivity in ELDerawi’s [38] experiment. Our previous study found that serum Ca decreased under DPN [8]. Diabetes damage signaling of Ca in axons, which is the main reason leading to diabetic neurodegeneration [39–41]. Disturbed homeostasis of Ca^2+^ in diabetic patients could have resulted from changed bone metabolism, altered dietary absorption, impaired organelle dysfunction [42], and endoplasmic reticulum stress [43, 44] of metabolic organs such as the liver and adipose tissue. Higher urinary Mg correlates positively with urinary Ca [45], resulting from decreased renal tubular reabsorption [8]. Ca^2+^-containing supplementation has been deemed effective in clinical experiments to improve diabetic nephropathy [46], bone metabolic reverse [47], and cardiovascular diseases [48]. Observations have been substantiated by intervention that relevant supplementation-improved glycated hemoglobin in T1D patients [49, 50]. Low serum Zn is seen in diabetes patients [9, 26], and Luo’s [26] experiment showed that low serum Zn was associated with diabetic microvascular complications. Zn is a protective element, acting as an antioxidant by protecting sulfhydryl groups of various proteins and enzymes against free radical damage [51]. Its reduction was on account of eliminated effect of antioxidation and free radicals scavenging, noticing oxidation stress is one of significant mechanisms of DPN [52]. Zinc supplementation could prevent disease progression to diabetes by controlling blood glucose and insulin resistance while improving β-cell function [53]. Diabetic patients are with lower serum Cr [54, 55], while many trials have confirmed that Cr supplementation altered high glucose status [56, 57] and slowed progression of complications [57–59]. The mechanism could be Cr directly bound with insulin and prolonged its duration of action plus antioxidative stress role [56]. Low Cr may attribute to increased loss plus decreased absorption [60], in that metabolic control system in T1D actually need additional Cr [61] while it cannot be utilized and eliminated through urination [62]. Elements concentration in urine may also be affected by high urinary excretion [9], osmotic change, etc.

### Cluster Analysis and Factor Analysis of Serum and Urine Elements in Control and DPN Patients

Cluster analysis is an exploratory method. The concept is to classify variables on characteristics so that the ones in the same category own the highest homogeneity as possible, whereas the different categories present the highest possible heterogeneity. The more intuitive explanation is that indicators are divided apart according to “distance”. Choice of specific algorithms depends mostly on variable type, sample size, and research purpose [65]. Factor analysis is a mean of solving multivariate association. As a simplification method, it classifies potential “categories” by decomposing the original information and combines them into several independent “factors” that reflect the overall information to represent several types of highly correlated variables. This method preserves the original variable information as much as possible and could be applied to explore the intrinsic structure of variables. The two methods seem to have opposite principles, but they actually complete each other in a certain sense.

Elements relate to each other in the human body, which has been proven by previous researches [8, 9, 20] and studies [10]. Factor analysis becomes applicable consequently. In summary, this study refers to Badran’s [10] research on selection of statistical models and feasibility for such subject.

The results showed that serum Cr, Se, and Fe were clustered in both groups and association of Mg and Ca in serum and urine of the DPN group was stronger. Similarly, factor analysis stated serum Cr, Se, and Fe were all correlated with factor 1, indicating that the three elements do relate to each other under both healthy and diseased status. That might be due to their common effects on insulin sensitivity [66–68] and oxidative stress [14, 69]. Serum Mg was strongly associated with factor 2 in the DPN group comparing to its relationship with factor 3 in control. That demonstrated Mg had greater influence for the DPN group, which might reveal as significant elevation of Mg under diabetic status [7, 8]. Table 3 presented the obvious correlation of serum Mg and Ca in DPN [8, 20] strongly interacted with factor 3, from the fact that product of Mg and Ca is quite fixed [8, 20]. Urinary Se correlated with Mg and Ca negatively under DPN, we had not found any logical evidence but supposed that this might due to the peroxidation state of diabetes based on the characteristic function of these antioxidant elements. Though positively correlated with factor 3, serum, Cu was less important for the DPN group. Badran [10] assumed that Cu was negatively associated with factor 1 and attributed that factor to metabolic discrepancy. The above conclusions had been considered for a specific population.

We had not observed the tendency to change of urinary elements. However, there were few likely studies indeed. To our knowledge, this is the first study to use multivariable statistical methods for discussion of urinary elements in diabetes. Although there are still some defects and objective obstacles, the whole design was unique. The results were obscure due to many possible factors. One is no reliable evidence for the selection of random urine specimens. However, different classification and factors of urine elements of two groups were extremely unlike, and the causes we supposed were as follows: impact on excretion rate due to multiple forms of various elements, charge, molecular weight and other components, etc. and great variation of physical and chemical alternation of urine. Palmer [70] believed that ion disorders in diabetic patients might be attributed to osmotic fluid transfer caused by hyperglycemia as well as fluid shortage because of diuresis, referring to permeability effecting ions’ flow.

Limitations of these experiments are the following: (1) Because of small sample size, there still remains uncertainty as to the results in the present study. (2) We use random urine in the study. However, 24 h urine specimen is a better choice. (3) Factor analysis may be weak for nonlinear model as the relationship could be nonlinear. (4) Practical theories on urinary elements remain unclear. However, the results are logical and consistent with current clinical trials. (5) Concentration of elements in urine may be determined by factors as dietary, absorption efficacy and kidney function, etc.

## Conclusion

Multivariate statistical methods could reduce the number of variables, integrate information, and explore their internal correlation. Cluster analysis’s principal is the homogeneity of variables. Factor analysis decomposes original information and recombines into factors representing several related variables. Our study confirmed that serum elements altered significantly in DPN against those of healthy population with reasonable mechanisms. Transformation medicine could be applied into clarification and treatment of disease. It is suggested that the future research and regimens adopt grouping model, that is putting similar elements together in a big picture or discussing relationship within them separately.
